# Supplementary material for: Dietary Pattern Influences Gestational Weight Gain: Results from the ProcriAr Cohort Study—São Paulo, Brazil
Source: Nutrients. 2022 Oct 21;14(20):4428. doi: 10.3390/nu14204428 (PMC9607592; doi:10.3390/nu14204428)
Supplement: Supplementary file 1 [file nutrients-14-04428-s001.zip › supplementary Table S1.pdf]

Supplementary Table S1: Description of food groups and average intake per day for each trimester and for the set of trimester in pregnant women. ProcriAr study (n = 385) - São Paulo/Brazil,2011-2013

**Food Groups**	**Grouping description**	**1º T**			**2ºT**			**3ºT**			**Set of trimester**		
		Intake (%)	Mean (g/day)	95%CI	Intake (%)	Mean (g/day)	95%CI	Intake (%)	Mean (g/day)	95%CI	Intake (%)	Mean (g/day)	95%CI
Beans and Lentils	Preparations or dishes with beans and/or lentils	63.0	46.5	43.3- 49.8	68.4	55.3	52.5-58.0	68.1	53.1	49.8- 56.4	66.5	52.9	50.3-55.5
Butter or margarine	Butter or margarine (regular and salted)	51.6	11.8	11.0-12.6	56.1	14.2	13.3-15.2	53.6	14.7	13.6-15.8	53.8	13.8	12.9-14.6
Cakes and Cookies	Simple cakes; Bakery with filling; Cookies; Brownies; sweet roll	18.7	15.2	13.6-16.8	29.1	25.4	23.0- 27.7	31.2	29.7	27.8-31.6	26.3	21.2	19.4- 23.0
Cereal and *Farofa*	Cereal ready to eat and Oats; Farofa, cassava or corn (flour)	18.3	11.9	10.3- 13.5	20.6	11.7	10.6-12.7	21.2	13.1	10.7-15.4	20.0	11.0	9.6-12.4
Chocolate (powder)	Chocolate flavours; milk, mixtures and milk drinks; Ovomaltine - dry mix, Chocolate Malt	27.4	14.4	12.1-16.7	30.5	15.7	13.7-17.8	29.3	15.7	13.3-18.1	29.1	15.6	13.7-17.5
Coffee sweetened	Coffee, regular (caffeinated), made from ground; cappuccino, prepared from recipe or restaurant, regular, without flavoured syrup	47.9	61.8	55.8-67.7	52.3	69.8	63.6-76.0	50.3	68.4	62.4-74.4	50.2	65.9	60.4-71.4

Supplementary Table S1: Description of food groups and average intake per day for each trimester and for the set of trimester in pregnant women. ProcriAr study (n = 385) - São Paulo/Brazil,2011-2013

Food Groups	Grouping description	1º T			2ºT			3ºT			Set of trimester		
		Intake (%)	Mean (g/day)	95%CI	Intake (%)	Mean (g/day)	95%CI	Intake (%)	Mean (g/day)	95%CI	Intake (%)	Mean (g/day)	95%CI
Crackers	Crackers butter regular; sprinkle cookies; cookies and bars butter plain	24.4	11.28	9.9- 12.7	24.5	12.52	10.8-14.3	22.5	10.6	9.5-11.6	23.8	10.9	9.9-11.9
Desserts and sweets	Ice cream and frozen desserts, milkshake; desserts with fruits and jelly; Candy, lollipop, whipped cream, coconut milk, condensed milk, chocolate, ice cream and bakery desserts - sweet pies, pudding, mousse	85.1	66.4	62.4-70.5	86.9	66.9	63.5-70.2	84.9	74.5	69.8-79.2	85.6	70.5	66.7-74.4
French bread	Croissant; bagels; white bread; French bread; Italian bread; baguette ;etc	69.2	57.2	54.6-59.8	70.9	60.7	57.7-63.8	68.6	60.9	57.8-63.9	69.9	60.5	58.0-63.1
Fruits	Orange; banana; apple; papaya; pineapple; mango; avocado; guava; persimmon; grape ;etc	55.7	149.7	141.2-158.2	48.1	129.7	119.1-140.3	45.7	122.8	112.1-133.6	42.3	134.0	124.5-143.6

Supplementary Table S1: Description of food groups and average intake per day for each trimester and for the set of trimester in pregnant women. ProcriAr study (n = 385) - São Paulo/Brazil,2011-2013

**Food Groups**	**Grouping description**	**1º T**			**2ºT**			**3ºT**			**Set of trimester**		
		Intake (%)	Mean (g/day)	95%CI	Intake (%)	Mean (g/day)	95%CI	Intake (%)	Mean (g/day)	95%CI	Intake (%)	Mean (g/day)	95%CI
Fruit smoothed and soy beverage	Various types of fruit smoothes with whole milk and soy milk drinks of various flavours	7.4	29.0	24.1-33.9	7.7	24.8	18.6-31.0	5.2	17.4	12.1-22.8	6.8	24.6	19.8-29.4
Lean meats	Lean meats and fish (Beef with vegetables, chicken, boiled egg and fish - roasted, cooked, soaked or fried)	44.9	61.4	57.7-65.1	40.4	58.3	54.1-62.6	41.7	61.9	57.5-66.3	42.3	59.5	56.0-62.9
Fried Meat, Chicken, eggs	Beef (roasted, cooked or soaked); Beef jerky; Fried beef and fried chicken; Fried egg or omelette; Offal (heart and liver)	48.6	58.0	56.5-59.4	54.5	69.1	64.7-73.4	52.9	72.4	66.4-78.4	52.0	67.1	64.0-70.2
Whole Milk and Yogurt	Whole milk; yogurt whole; yogurt with flavour	60.5	147.3	136.7-158.0	63.4	168.7	157.2-180.3	68.4	178.7	166.5-190.9	64.1	164.0	153.4-174.7

Supplementary Table S1: Description of food groups and average intake per day for each trimester and for the set of trimester in pregnant women. ProcriAr study (n = 385) - São Paulo/Brazil,2011-2013

**Food Groups**	**Grouping description**	**1º T**			**2ºT**			**3ºT**			**Set of trimester**		
		Intake (%)	Mean (g/day)	95%CI	Intake (%)	Mean (g/day)	95%CI	Intake (%)	Mean (g/day)	95%CI	Intake (%)	Mean (g/day)	95%CI
Mozzarella cheese	American cheese, process; Cheddar cheese, process cheese spread; Brie cheese; Mozzarella cheese, whole milk; Parmesan cheese, dry (grated), regular; provolone cheese, natural; Mozzarella cheese, whole milk;	16.2	5.0	4.3-5.7	14.9	4.2	3.6-4.9	13.9	3.2	2.6-3.8	15.0	4.0	3.5-4.5
Oil salad dressing	Dressing for salads; oil olive	33.9	2.6	2.4-2.9	34.7	2.4	2.2-2.6	32.5	2.4	2.3-2.6	33.7	2.3	2.1-2.5
Pasta	Pasta meatless and meat sauce; gnocchi; stuffed pasta	35.1	67.9	65.8-70.0	34.0	69.1	63.0-75.3	35.5	65.2	61.7-68.7	34.8	64.8	60.7-68.8
Pork and Frankfurters	Bacon regular; sausage pork; sausage smoked; pork fresh; pork steak	16.2	13.7	11.6-15.8	19.2	19.2	17.5-20.9	16.2	18.2	16.3- 20.1	17.2	14.3	12.6-16.0

Supplementary Table S1: Description of food groups and average intake per day for each trimester and for the set of trimester in pregnant women. ProcriAr study (n = 385) - São Paulo/Brazil,2011-2013

Food Groups	Grouping description	1º T			2ºT			3ºT			Set of trimester		
		Intake (%)	Mean (g/day)	95%CI	Intake (%)	Mean (g/day)	95%CI	Intake (%)	Mean (g/day)	95%CI	Intake (%)	Mean (g/day)	95%CI
Potato and cassava boiled	Potato, baked or boiled with skin or without skin ; sweet potato, baked or boiled; mashed potatoes, prepared from fresh; mashed potatoes, unknown if prepared from fresh or dehydrated; potato, roasted (includes Franconia); potato, au gratin, prepared from fresh	20.4	25.0	24.5-25.4	17.5	25.2	22.1-28.3	17.8	20.4	18.5-22.4	18.6	20.6	18.9-22.4
Processed Meat and Snacks	Hamburger, chicken nuggets, meatball, lunchmeats - ham, mortadella and salami; sandwiches - hot dog, hamburger; fried snacks, baked savoury, pizza and snacks	50.6	71.9	68.1-75.8	53.9	78.6	74.6- 82.7	52.1	78.9	74.5-83.4	52.2	75.9	71.5-80.2
Rice white	Preparations or dishes with white rice	85.5	146.1	139.3-152.9	84.0	157.4	150.6-164.1	82.6	157.7	150.3-165.1	83.0	156.5	150.2-162.7
Salt	Added salt	40.1	0.59	0.49-0.62	39.9	0.6	0.48-0.63	34.4	0.45	0.42-0.47	38.1	0.51	0.47-0.56

Supplementary Table S1: Description of food groups and average intake per day for each trimester and for the set of trimester in pregnant women. ProcriAr study (n = 385) - São Paulo/Brazil,2011-2013

Food Groups	Grouping description	1º T			2ºT			3ºT			Set of trimester		
		Intake (%)	Mean (g/day)	95%CI	Intake (%)	Mean (g/day)	95%CI	Intake (%)	Mean (g/day)	95%CI	Intake (%)	Mean (g/day)	95%CI
Condiments	Sauce cheese; ketchup regular; mustard; barbecue sauce; mayonnaise;	7.1	3.0	2.4-3.6	4.9	2.3	1.5-3.0	5.6	2.1	1.5-2.7	5.9	2.3	1.9-2.8
Soft Drinks	Cola regular; soda pop; non-cola regular; coca diet	39.0	155.8	142.7-168.8	44.8	194.2	180.1-208.3	44.5	228.2	216.4-240.0	42.8	187.3	174.5-200.1
Sweetened Juices	Artificial juice (sweetened), natural juice (sweetened)	50.9	159.6	142.9-176.4	53.0	149.0	129.4-168.5	52.2	178.3	159.8-196.9	52.0	167.6	146.7-188.4
Milk and fat-reducing derivatives	milk, 2% fat or reduced fat; milk, 1% fat or low fat; milk, skim, non-fat or fat free; cream cheese; ricotta pate	16.2	15.7	12.9-18.6	14.9	14.4	11.4-17.3	13.9	11.6	9.0-14.1	15.0	14.3	11.7-16.9
Sweetened tea	Green tea; herbs; black; matte; mixed herbal and dried fruit (sweetened)	6.4	14.4	10.0-18.9	5.6	13.2	8.9-17.5	6.0	14.4	10.1-18.7	6.0	16.1	12.7-19.4
Unsweetened juices	Artificial juice (unsweetened), natural juice (unsweetened)	16.1	60.4	52.2-68.6	14.7	58.1	49.2-67.0	16.4	73.6	65.2-82.0	15.7	59.6	52.1-67.1

Supplementary Table S1: Description of food groups and average intake per day for each trimester and for the set of trimester in pregnant women. ProcriAr study (n = 385) - São Paulo/Brazil,2011-2013

**Food Groups**	**Grouping description**	**1º T**			**2ºT**			**3ºT**			**Set of trimester**		
		Intake (%)	Mean (g/day)	95%CI	Intake (%)	Mean (g/day)	95%CI	Intake (%)	Mean (g/day)	95%CI	Intake (%)	Mean (g/day)	95%CI
Vegetables	Lettuce; spinach; watercress; kale; cabbage; cauliflower; tomato; carrot; eggplant; beets; chayote; pumpkin; cucumber; zucchini; onion	58.1	63.0	58.8- 67.1	54.2	56.2	52.4- 59.9	47.9	46.6	43.7-49.6	53.4	54.2	50.7- 57.7
Vinaigrette	Onion, tomato, pepper, oil, salt, vinegar and parsley	21.3	2.4	2.1-2.6	22.2	2.4	2.1-2.7	21.0	1.8	1.6- 2.1	21.5	2.1	1.8-2.3
Wheat bread and Brown Rice	Wheat bread, brown rice	5.3	4.6	2.9-6.2	5.8	5.1	3.7-6.6	6.6	6.0	4.4-7.7	5.9	5.4	4.3-6.5
