# Supplementary material for: Dietary Pattern Influences Gestational Weight Gain: Results from the ProcriAr Cohort Study—São Paulo, Brazil
Source: Nutrients. 2022 Oct 21;14(20):4428. doi: 10.3390/nu14204428 (PMC9607592; doi:10.3390/nu14204428)
Supplement: Supplementary file 1 [file nutrients-14-04428-s001.zip › supplementary Table S2.pdf]

Supplementary Table S2:
Factor loadings of food group's characteristic to the principal dietary components identified in each trimester in pregnant women. of the ProcriAr Cohort, São Paulo 2011-2013.

Food Groups	First Trimester			Second Trimester			Third Trimester		
	Fruits and	Brazilian	Western	Brazilian	Fruits and	Western	Fruits and	Western	Brazilian
Beans and Lentils	0.3420	0.4694			0.5439				0.3931
Butter or margarine		0.5792				-0.5925			0.6223
Chocolate (powder)									
Coffee sweetened		0.3619	-0.3068						0.4134
Rice white	0.4543	0.4843			0.6703		0.3431		0.3782
Crackers			-0.3003		0.3533			-0.3320	
French bread		0.6428				-0.3731			0.6283
Fruits				0.4344				-0.3763	
Lean meats									
Mozzarella cheese									
Oil salad dressing	0.7565			0.6230	0.3011		0.7949		
Pork and Frankfurters					0.3907			0.3901	
Potato and cassava boiled									
Processed Meat and Snacks			0.6237			0.5044		0.5042	
Salt	0.4510						0.7578		
Condiments			0.3559						
Soft Drinks			0.6640			0.3708		0.6885	
Fruit smoothed and soy beverage									
Sweetened Juices		-0.3184							
Sweetened tea									
Unsweetened juices									
Vegetables	0.7507			0.7327			0.7847		
Vinaigrette	0.4511				0.4741		0.5171		
Wheat bread and Brown Rice									-0.3428
Milk and fat-reducing derivatives									
Cookies e Cakes						0.3349		0.5009	
Desserts and sweets						0.3685			
Pasta									
Whole Milk and Yougurt						-0.3128			
Cereals and Farofa									
Fried Meat, Chicken and eggs		0.4043			0.4322				0.3384

Food groups presented had factor loadings -0.3 or 0.3 and were therefore used to describe each dietary pattern
